# Quantification of Exercise‐Induced Sarcomeric Damage in R349P Desmin Knock‐In Mice: A New Approach in Myofibrillar Myopathy Research

**DOI:** 10.1111/nan.70038

**Published:** 2025-09-14

**Authors:** Christian Holtzhausen, Dorothea Schultheis, Carolin Berwanger, Julia Schuld, Ursula Schlötzer‐Schrehardt, Marion Riehl‐Nestler, Sabrina Batonnet‐Pichon, Alain Lilienbaum, Esther Mahabir, Peter F. M. van der Ven, Dieter O. Fürst, Rolf Schröder, Christoph S. Clemen

**Affiliations:** ^1^ Institute of Neuropathology Universitätsklinikum Erlangen, Friedrich‐Alexander‐Universität Erlangen‐Nürnberg (FAU) Erlangen Germany; ^2^ Institute of Aerospace Medicine German Aerospace Center Cologne Germany; ^3^ Institute of Vegetative Physiology, Medical Faculty University of Cologne Cologne Germany; ^4^ Institute for Cell Biology University of Bonn Bonn Germany; ^5^ Department of Ophthalmology Universitätsklinikum Erlangen, Friedrich‐Alexander‐Universität Erlangen‐Nürnberg (FAU) Erlangen Germany; ^6^ Comparative Medicine, Center for Molecular Medicine Cologne, Faculty of Medicine and University Hospital Cologne Cologne Germany; ^7^ Basic and Translational Myology, Unit of Functional and Adaptive Biology Université Paris Cité/CNRS UMR 8251 Paris France; ^8^ Institut Cochin, Université Paris Cité, INSERM U1016, CNRS Paris France

**Keywords:** desmin, desminopathy, exercise‐induced muscle damage, FLNC, myofibrillar myopathies, quantitation of sarcomeric lesions, treadmill exercise, xirp

## Abstract

**Aims:**

The classical morphological hallmarks of the clinically and genetically diverse group of human myofibrillar myopathies are signs of myofibrillar degeneration and desmin‐positive protein aggregates. The local sarcomeric enrichment of filamin‐C and xin actin‐binding repeat‐containing proteins 1 and 2 (xirp 1 and xirp 2) is a marker of myofibrillar damage. This work aimed to (i) address filamin‐C‐ and xirp 1/2‐positive sarcomeric lesions in desminopathy mouse models, (ii) develop an approach to quantifying xirp 1/2‐positive sarcomeric lesions, and (iii) study the effects of acute physical exercise on sarcomeric lesion formation in R349P desmin knock‐in mice, which are a model of human R350P desminopathy.

**Methods:**

Sarcomeric lesions were visualised by xirp 1/2 and filamin‐C immunofluorescence in soleus muscles from R349P and R405W desmin knock‐in, desmin knock‐out and W2711X filamin‐C knock‐in mice. The open‐source software QuPath was used to analyse xirp 1/2 confocal immunofluorescence images of soleus muscle from nonexercised and treadmill‐exercised R349P desmin knock‐in mice.

**Results:**

Filamin‐C and xirp 1/2 stained muscles revealed the presence of congenerous sarcomeric lesions in heterozygous and homozygous R349P and R405W desmin knock‐in, homozygous desmin knock‐out and heterozygous and homozygous W2711X filamin‐C knock‐in mice. Quantitative analysis of R349P desmin knock‐in mice showed (i) significantly more lesions in nonexercised heterozygous mice, with an even more pronounced increase in homozygous animals, as compared to wild‐type, and (ii) a significant increase in sarcomeric lesions per mm^2^ in heterozygous mice and wild‐type siblings subjected to strenuous treadmilling.

**Conclusions:**

A QuPath workflow to quantify sarcomeric lesions using xirp 1/2 immunofluorescence images showed augmented densities of lesions in R349P desminopathy mice and after treadmill exercise. Eccentric and high‐intensity physical activity may exhibit a disease‐promoting effect on skeletal muscles in desminopathy.

## Summary

- This study developed a novel approach to quantify sarcomeric lesions in cross‐sections of skeletal muscle tissue at the light microscope level.
- This study addresses xirp and filamin‐C‐positive sarcomeric lesions in soleus muscles from R349P and R405W desminopathy, desmin knock‐out and W2711X filaminopathy mice
- Strenuous treadmill exercise significantly increases sarcomeric lesion burden in R349P desminopathy mice.
- Eccentric and high‐intensity physical exercise may exacerbate sarcomeric damage in human desminopathies

## Graphical abstract

This study presents a new approach to quantifying sarcomeric lesions in cross‐sections of skeletal muscle tissue at the light microscope level. Using R349P‐desmin knock‐in mice, a model for the human R350P desminopathy, strenuous treadmill exercise was found to lead to a significant increase in xirp 1/2‐positive sarcomeric lesions in the soleus muscle. The obtained data suggest that eccentric and high‐intensity physical exercise may exacerbate sarcomeric damage in human desminopathies.

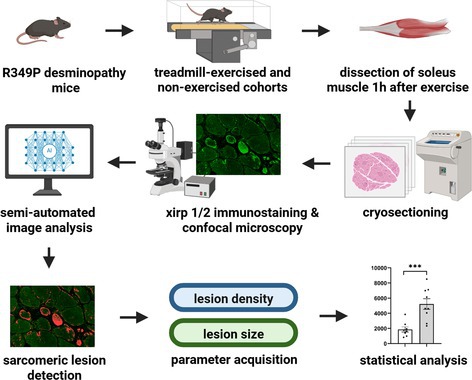

## 1 Introduction

The clinically and genetically diverse group of myofibrillar myopathies represents a numerically significant subgroup of human protein aggregate myopathies [1]. Over the last three decades, multiple gene defects causing myofibrillar myopathy have been identified, resulting in autosomal‐dominant, autosomal recessive and X‐linked inheritance [2]. The spectrum of affected genes comprises genes encoding sarcomeric proteins (*FLNC*, *LDB3*, *MYOT*, *BAG3*, *TTN*, *FHL1*), extrasarcomeric cytoskeletal proteins (*DES*, *PLEC*, *SVIL*) and constituents of the protein quality control machinery (*CRYAB*, *DNAJB6*, *HSPB8*, *KLCH24*, *VCP*) [1, 2, 3]. Irrespective of the individual gene defect, the striated muscle pathology of myofibrillar myopathies is characterised by desmin‐positive sarcoplasmic and subsarcolemmal protein aggregates and degenerative changes of the myofibrillar apparatus [1, 3, 4, 5]. These degenerative changes range from small myofibrillar disruptions, such as focal Z‐disc broadening and streaming, to the formation of rods and ultimately the complete dissolution of myofibrils [4, 6, 7]. However, none of these changes is specific to myofibrillar myopathies, as they can be found in a wide variety of neuromuscular disorders [8]. Moreover, Z‐disc streaming can even be observed in normal human skeletal muscle in response to physical exercise and eccentric contractions, referred to as exercise‐induced muscle damage or delayed onset muscle soreness [9, 10, 11, 12, 13, 14, 15]. Focal disruption of Z‐discs affecting single to multiple adjacent myofibrils has also been reported in W2711X filamin‐C knock‐in mice, which harbour the most frequently occurring *FLNC*‐associated myofibrillar myopathy‐causing human W2710X filamin‐C mutation. Notably, the occurrence of so‐called sarcomeric microlesions (spanning one to five sarcomeres) or macrolesions (spanning more than five sarcomeres) increased in response to strenuous treadmill running in heterozygous and homozygous W2711X filamin‐C knock‐in mice, indicating that expression of mutant filamin‐C or lack of wild‐type filamin‐C negatively affects myofibrillar stability in skeletal muscle tissue [16, 17]. Since both filamin‐C and the xin actin‐binding repeat‐containing proteins 1 and 2 (xirp 1, xirp 2) rapidly localise to segments of myofibrillar damage, antibodies against these proteins can visualise sarcomeric lesions in immunofluorescence microscopy of skeletal muscle sections [16, 17, 18, 19, 20, 21, 22, 23]. In contrast to the visualisation of sarcomeric damage in electron micrographs, immunostaining with filamin‐C or xirp 1/2 antibodies therefore generally allows the quantitation of the number and size of the sarcomeric lesions in normal and diseased skeletal muscle tissues.

In the present study, we investigated and quantified the presence of xirp 1/2‐ and filamin‐C‐positive sarcomeric lesions in skeletal muscle tissue from mouse models of desminopathies. We analysed heterozygous and homozygous R349P desmin knock‐in mice [24] and R405W desmin knock‐in mice [25, 26], which serve as disease models for the human R350P and R406W desminopathies, respectively. For comparison, we used the W2711X filamin‐C knock‐in mice [16, 17] as well as desmin knock‐out mice [27, 28], a model for the rare autosomal recessive desminopathy subform without desmin expression, and corresponding wild‐type siblings. We also investigated the influence of strenuous treadmill running on sarcomeric lesion formation in wild‐type and heterozygous and homozygous R349P desmin knock‐in mice. To quantify xirp 1/2‐positive sarcomeric lesions, we established a workflow for the digital analysis of immunofluorescence images of murine soleus muscle sections using the open‐source application QuPath [29].

## 2 Materials and Methods

### 2.1 Mouse Models

Four previously characterised desminopathy and filaminopathy mouse models were used in this study: p.R349P desmin knock‐in mice (http://www.informatics.jax.org/allele/MGI:5708562, B6J.129Sv‐*Des* ^tm1.1Ccrs^/Cscl, [24]); p.R405W desmin knock‐in mice (http://www.informatics.jax.org/allele/MGI:6382571, C57BL/6 N‐*Des* ^tm1.1Allb^/Allb, [25]), desmin knock‐out mice (http://www.informatics.jax.org/allele/MGI:2159584, B6J.129S2/Sv‐*Des* ^tm1Cba^/Cscl, [27]); and p.W2711X filamin‐C knock‐in mice (http://www.informatics.jax.org/allele/MGI:5907163, B6J.B6‐*Flnc* ^tm1.1Rsdf^/Cscl, [16]). All mice were housed in individually ventilated cages under specific opportunistic pathogen‐free conditions in a standard environment with unrestricted access to food and water. Health monitoring was done according to the recommendations of the Federation of European Laboratory Animal Science Associations [30]. Mice were handled in accordance with (i) the German Animal Welfare Act (Tierschutzgesetz) as well as the German Regulation for the protection of animals used for experimental or other scientific purposes (Tierschutz‐Versuchstierverordnung) or (ii) the French regulations for animal experimentation. All procedures and investigations for the purpose of this study were approved by (i) the governmental office for animal care (Landesamt für Natur, Umwelt und Verbraucherschutz North Rhine‐Westphalia, Recklinghausen, Germany [reference number 84‐02.04.2016.A421]) or (ii) the University Paris Diderot local committee (authorisation number CEB‐16‐2016/2016041216476300).

### 2.2 Treadmill Exercising of R349P Desmin Knock‐In Mice

A computer‐controlled, motorised treadmill device with six running lines (TSE Treadmill System; TSE Systems GmbH, Berlin, Germany) equipped with an automatic ‘air‐puff’ system was employed for acute, strenuous exercising of R349P desmin knock‐in mice (heterozygous, 12x female [6x run, 6x no run control], 6x male (3x run, 3x no run control); homozygous, 13x female [7x run, 6x no run control], 9x male [5x run, 4x no run control]) and wild‐type siblings (11x female [6x run, 5x no run control)], 6x male [3x run, 3x no run control]) (Tables 1 and S1). Table 1 provides an overview of the six groups of mice with the number of animals, sex, mean and median age and age range. Table S1 additionally provides details on each mouse and the image analysis data. Running belt speed settings were 0.1 m/s at start with a continuous increase to a maximum of 0.7 m/s within a period of 60 min. The untrained mice usually got exhausted after approximately 30 min at a belt speed of 0.35 m/s at the latest. Mice were sacrificed by cervical dislocation 1 h after completion of the treadmill running, and muscles were dissected, snap‐frozen in liquid nitrogen‐cooled isopentane and stored at −80°C until analysis.

**TABLE 1:** Overview of the six groups of mice.

Experimental group	Number of animals	Sex (m/f)	Mean age in months	Median age in months	Age range in months
**DKI, WT, no exercise**	**8**	**3/5**	**15**	**13**	**26**
**DKI, WT, exercise**	**9**	**3/6**	**9**	**6**	**15**
**DKI, Het, no exercise**	**9**	**3/6**	**21**	**24**	**24**
**DKI, Het, exercise**	**9**	**3/6**	**9**	**6**	**15**
**DKI, Hom, no exercise**	**10**	**4/6**	**22**	**24**	**10**
**DKI, Hom, exercise**	**12**	**5/7**	**11**	**8**	**16**

*Note:* Table providing basic data (number of animals, sex, mean and median age, age range) for the groups of wild‐type and heterozygous and homozygous R349P desmin knock‐in mice assigned to control or strenuous exercise (run).

### 2.3 Electron Microscopy

A standard electron microscopy protocol was used. Briefly, murine soleus muscle samples were fixed in 2.5% glutaraldehyde in 0.1 M of phosphate buffer, pH 7.2, postfixed in 0.5% buffered osmium tetroxide, dehydrated in graded alcohol concentrations and embedded in Durcupan resin (Fluka, Switzerland). Ultrathin sections were prepared (Ultracut S; Leica, Germany), stained with uranyl acetate and lead citrate and examined with a Zeiss LEO 906E (Carl Zeiss GmbH, Oberkochen, Germany) transmission electron microscope.

### 2.4 Indirect Immunofluorescence Staining

Cryopreserved soleus/gastrocnemius muscle packages were sectioned until the soleus part reached its maximum diameter. Sections from this part of the specimen were used for H&E staining to ensure optimal morphological quality of the soleus muscle tissue. Three to five serial cryosections of 5‐μm thickness per slide were fixed in −20°C cold acetone for 10 min and air‐dried. Slides were then blocked using 1x PBS supplemented with 10% FCS, 1% goat serum and 0.01% NaN_3_ and incubated for 60 min on an orbital shaker. Primary and secondary antibodies were diluted in 1x PBS containing 10% FCS, and the slides were incubated for 60 min in a light‐protected wet chamber at room temperature. After each incubation step, the samples were washed in 1x PBS for 3 times 5 min on an orbital shaker. Finally, coverslips were mounted on slides using Mowiol/DABCO. The stained section with the fewest artefacts and the best‐preserved morphology was selected from each slide for microscopy and image analysis. Images were acquired using a confocal laser‐scanning microscope (LSM780, Carl Zeiss, Oberkochen, Germany) with a Plan‐Apochromat 40x/1.4 Oil DIC M27 objective.

### 2.5 Antibodies

Primary antibodies and dilutions used the following: Anti‐filamin‐C (RR90, mouse monoclonal, IgA isotype, 1:50 [31]); Anti‐titin (T12, epitope close to the Z‐disc, mouse monoclonal, 1:20 [32]); and Anti‐xin actin‐binding repeat‐containing proteins 1 and 2 (#7700, rabbit polyclonal, 1:750 [19]); in this reference, a purified bacterially expressed recombinant peptide comprising xin repeats 2–10 of murine xin actin‐binding repeat‐containing protein 2 (xirp 2, *Xirp2*) was used for immunisation and detection of xirp 2; this antiserum also detects xin actin‐binding repeat‐containing protein 1 (xirp 1, *Xirp1*) (Figure S1); the sequence of the immunisation peptide was as follows: aa335‐LEWDEILKGEVQSIRWIFENQPLDSINHGSTDEGYTSKGIADQELIAGSDVKYTTWMFETQPIDALGIPSAGTEGNTEKIPELARGDVYTARWMFETRPLDSMNKMHECQEETASTLTKDITGGDVKTVRYMFETQQLDQLGQLHSVDELNLLQLRSELKEIKGNVKRSIKCFETQPLYVIRDGSGQMLEIKTVQREDIEKGDVRTARWMFETQPLDTINKDITEIKVVRGISMEENVKGGVSRAKWLFETQPLEKIKEESGEAVLKTEAVIGTDVSKKCWMFETQPLDILKDSPDTDSVSPEERIGGDVKTTKHLFETLPIEALKDSPDIGK–667 (Uniprot Q4U4S6‐1); the clone to express the recombinant protein for immunisation will be shared upon request.

Secondary antibodies and dilutions were used: goat‐anti‐rabbit‐Alexa Fluor 488, 1:100 (Thermo Fisher (Invitrogen), A‐11034); goat‐anti‐rabbit Alexa Fluor 555, 1:100 (Thermo Fisher (Invitrogen), A‐21429); goat‐anti‐mouse‐IgA‐Alexa Fluor 488, 1:100 (Biozol Diagnostica/Southern Biotech, SBA‐1040‐30); and goat‐anti‐mouse‐Alexa Fluor 647, 1:100 (Thermo Fisher (Invitrogen), A‐21236).

### 2.6 Software‐Based, Semi‐Automatic Sarcomeric Damage Quantification

The open‐source digital pathology application QuPath (v.0.3.0, https://qupath.github.io, [29]) was used to train a binary pixel classifier in order to enable semi‐automatic counting and measurement of xirp 1/2‐positive sarcomeric lesions in stitched immunofluorescence images from tile scans of soleus muscle sections. Primary readout parameters were (i) cross‐sectional muscle area of interest in μm^2^, (ii) total lesion count, (iii) mean lesion size in μm^2^ and (iv) number of macrolesions; further calculated parameters were (v) lesion density in lesions/mm^2^, (vi) fraction of macrolesions per total lesions in percent and (vii) macrolesion density in macrolesions/mm^2^.

To generate a training dataset, cryosections of gastrocnemius/soleus muscle bundles were subjected to a standardised indirect immunofluorescence staining protocol in order to minimise inter‐batch immunostaining variability. Tile‐scan images of representative samples derived from each genotype were recorded using a confocal laser‐scanning microscope (LSM780, Carl Zeiss, Oberkochen, Germany) with a Plan‐Apochromat 40x/1.4 Oil DIC M27 objective. Acquisition settings were standardised based on a benchmark image and kept identical for the entire set of samples analysed. Lesion quantification was restricted to the soleus muscle in all samples, and this region of interest (ROI) was defined using the brush tool in QuPath, thereby excluding artefacts. Manual annotations are required for training of pixel classifiers in QuPath, so that at first, the following two classes were defined: Class 1 (positive/lesion) and Class 2 (ignore/no lesion). Individual sarcomeric lesions were annotated as Class 1, while areas containing background, noise signal, and connective tissue were defined as Class 2. Within each ROI of the training dataset, a minimum of 100 objects were manually annotated for each class, with an equal number of annotations for both classes. After an initial performance evaluation of the different algorithms and options available in QuPath applied to the training data, the following settings were chosen: Classifier—Artificial neural network (ANN_MLP), Resolution—Full (0.13 μm/px), Features—Default multiscale features and Output—Classification. Using these settings, the pixel classifier was trained iteratively, and iterations were saved after each training image for subsequent evaluation of performance. Classification performance was considered optimal after four training images, with the resulting iteration providing the best compromise between overall detection sensitivity and false positives (Figure 3A).

For final analysis, all samples were classified using the same iteration of the pixel classifier described above. Investigator input was limited to setting the soleus muscle ROI. The investigator was blinded to the treadmill intervention, but blinding for genotype was not possible due to obvious morphological and immunostaining differences between samples derived from wild‐type, heterozygous and homozygous animals. Automated pixel classification was performed and resulted in the above readout parameters (Figure 3B); the minimum detection size was set to ≥ 0.01 μm^2^. The output was stored and used for statistical analysis.

### 2.7 Statistical Analysis

Statistical analysis was performed using GraphPad Prism version 9.5.1. Datasets were tested for normal distribution using the Anderson–Darling test; in the ‘HET no run’ group, the values for ‘lesion density’ and ‘macrolesion density’ did not pass the normality test. For comparisons between multiple datasets, significant differences in standard deviation between datasets were assessed using the Bartlett and the Brown–Forsythe tests. Dataset comparisons of normally distributed data with similar standard deviation were performed using ordinary one‐way ANOVA, and means of each dataset were compared using post hoc Tukey's multiple comparison test. Where standard deviations differed significantly, which was the case for the ‘macrolesion density run’ groups, the Brown–Forsythe and the Welch ANOVA tests were used, followed by Dunnett's T3 multiple comparisons test. Dataset comparisons of nonnormally distributed data were performed using the Kruskal–Wallis test, followed by the Bonferroni–Holm corrected Mann–Whitney test. For additional comparisons between two specific datasets of interest, two‐tailed Student's *t*‐test with or without Welch's correction was used for normally distributed data, depending on differences in variance between groups, and for nonnormally distributed data, the Mann–Whitney U test was used. The *p*‐values < 0.05 were considered significant. All data are expressed as mean ± SEM.

## 3 Results

### 3.1 Sarcomeric Lesions in W2711X Filamin‐C Knock‐In, R349P Desmin Knock‐In, R405W Desmin Knock‐In, and Desmin Knock‐Out Mice

**FIGURE 1:**
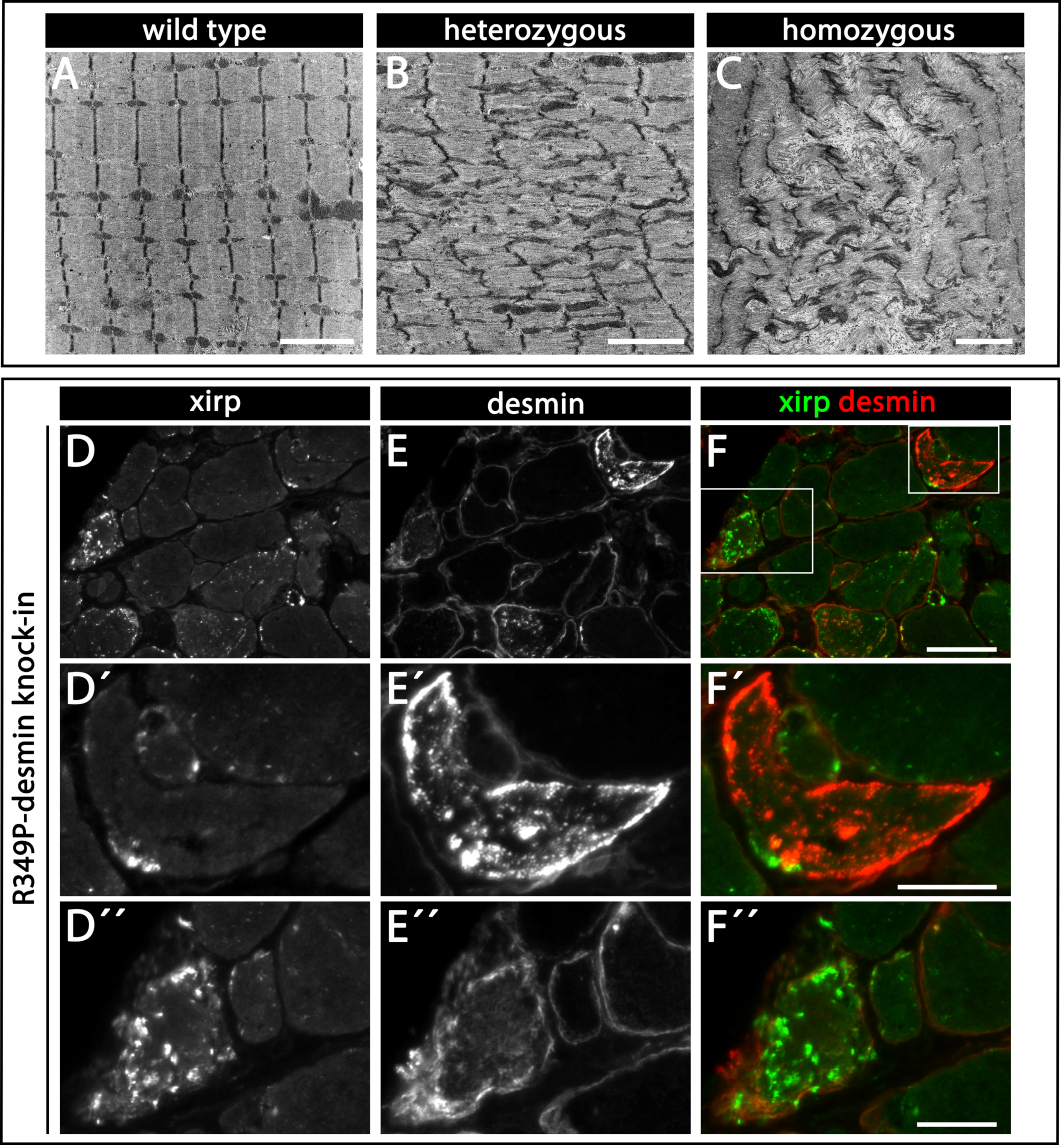
Sarcomeric pathology in R349P desmin knock‐in mice visualised by electron and xirp 1/2 immunofluorescence microscopy. (A) Regular, highly ordered and aligned myofibrils in electron micrographs of soleus muscle derived from wild‐type animals. (B, C) Ultrastructural illustration of sarcomeric pathology in soleus muscle of heterozygous (B) and homozygous (C) R349P desmin knock‐in mice comprising Z‐disc streaming and focal myofibrillar dissolution, respectively. (D–F″) Xirp 1/2 (xirp) and desmin double‐immunofluorescence staining of cross‐sections of soleus muscle from homozygous R349P desmin knock‐in mice. Overlays (right columns), xirp 1/2 (xirp) in green and desmin in red. Higher magnifications of desmin‐positive, xirp 1/2‐negative protein aggregates (D′–F′) and xirp 1/2‐positive, desmin‐negative sarcomeric lesions (D″–F″). Scale bars in A–C, 2.5 μm; scale bar in F, 50 μm; scale bars in F′ and F″, 20 μm.

Myofibrillar pathology, a classical hallmark of desminopathies, filaminopathies and other forms of myofibrillar myopathies, is usually visualised by means of transmission electron microscopy in longitudinally oriented areas of skeletal muscle tissue. Concurrently, soleus muscles from heterozygous and homozygous R349P desmin knock‐in mice exhibited a sarcomeric pathology comprising Z‐disc streaming and focal myofibrillar dissolution (Figure 1A–C and [24]), indistinguishable from the sarcomeric pathology observed in heterozygous and homozygous W2711X filamin‐C knock‐in [16, 17], heterozygous and homozygous R405W desmin knock‐in [26] and desmin knock‐out mice [27, 33, 34]. At the light microscope level, the Z‐disc‐associated proteins filamin‐C and xin actin‐binding repeat‐containing proteins 1 and 2 (xirp 1 and xirp 2) proved to be sensitive markers of sarcomeric lesions, both in normal and filaminopathy muscle tissues [16, 17, 18, 19, 20, 21, 22, 23]. To rule out the possibility that xirp 1/2 also stains desmin‐positive protein aggregates, we performed xirp 1/2 and desmin double‐immunostains of soleus muscle cross‐sections from homozygous R349P desmin knock‐in mice (Figure 1D–F). These double‐stains revealed that desmin‐positive subsarcolemmal and sarcoplasmic protein aggregates generally were negative for xirp 1/2 (Figure 1D′–F′), and vice versa, the xirp 1/2‐positive lesions were negative for desmin (Figure 1D″–F″).

**FIGURE 2:**
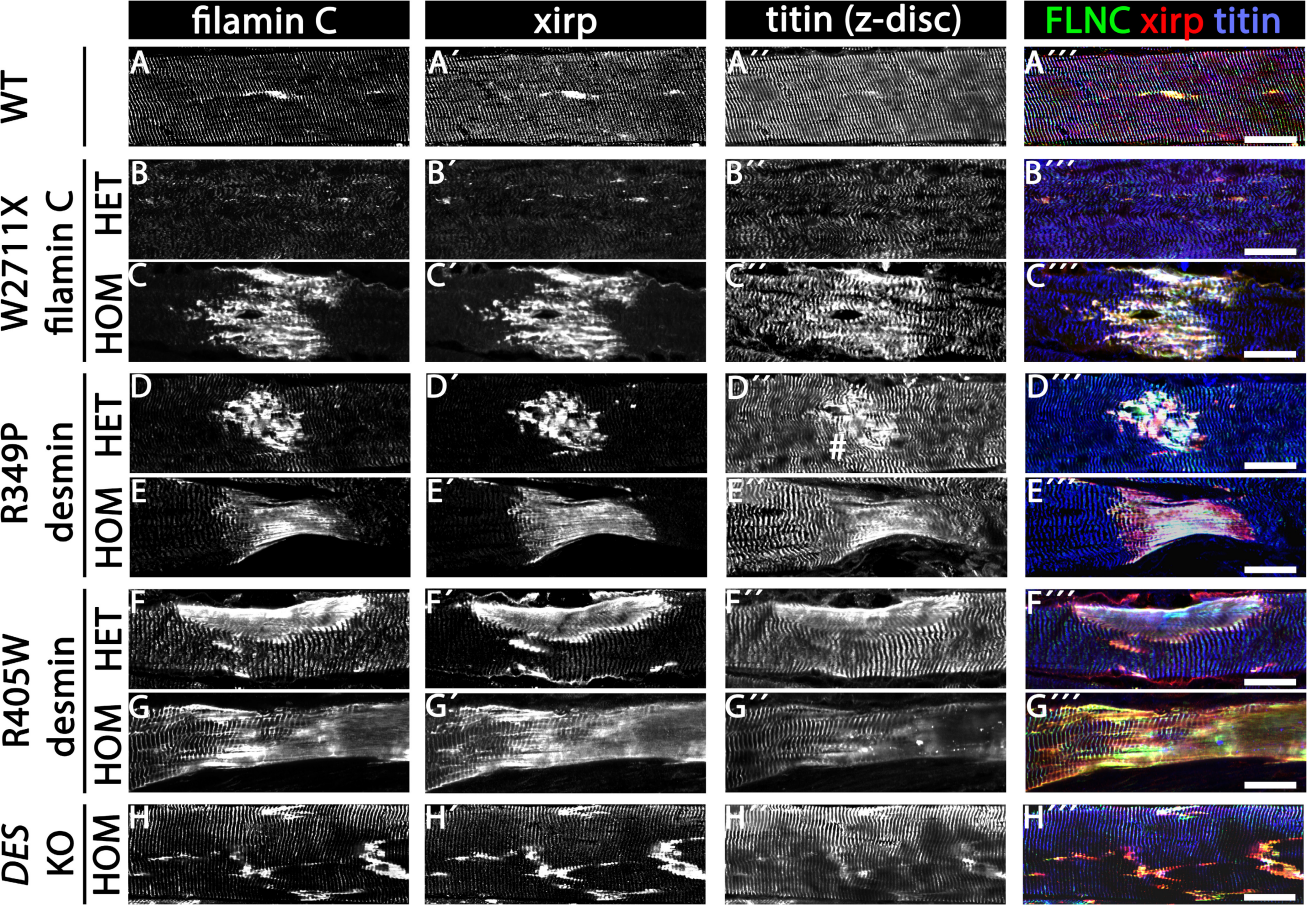
Sarcomeric lesions in longitudinal sections of soleus muscle derived from filaminopathy and desminopathy mouse models. (A–H) Immunofluorescence images illustrating the presence of filamin‐C (A–H) and xirp 1/2 (xirp) (A'–H′) positive sarcomeric microlesions and macrolesions in wild‐type siblings (A), heterozygous (B) and homozygous (C) W2711X filaminopathy, heterozygous (D) and homozygous (E) R349P desminopathy, heterozygous (F) and homozygous (G) R405W desminopathy and homozygous (H) desmin knock‐out mice. Titin Z‐disc epitope to visualise myofibrils (A″–H″). While sarcomeric microlesions typically span between 1 and 5 sarcomeres, macrolesions extend over larger areas and across adjacent myofibrils [16]. Note (i) that microlesion and macrolesion occur in the same fibres, (ii) that the images from heterozygous W2711X soleus muscle (B) only illustrate microlesions and (iii) that wild‐type soleus muscle (A) also harbours occasional lesions. Overlays (A′′′–H′′′), filamin‐C in green, xirp 1/2 (xirp) in red and titin Z‐disc epitope in blue. Scale bars, 25 μm.

We then analysed longitudinal sections of soleus muscle from wild‐type, heterozygous and homozygous W2711X filamin‐C knock‐in, heterozygous and homozygous R349P desmin knock‐in, heterozygous and homozygous R405W desmin knock‐in and homozygous desmin knock‐out mice. Filamin‐C, xirp 1/2 and titin immunofluorescence staining showed the presence of filamin‐C and xirp 1/2‐positive sarcomeric lesions in all genotypes (Figure 2A–H″). While the images derived from wild‐type and heterozygous W2711X filamin‐C knock‐in muscles illustrated only sarcomeric microlesions (Figure 2A,B″), all other genotypes displayed a mixture of sarcomeric microlesions and macrolesions (Figure 2C–H"). The largest lesions spanning the entire diameter of a muscle fibre and associated with fibre constriction were observed in homozygous R349P desmin knock‐in mice (Figure 2E–E″). In addition, the xirp 1/2 signal was markedly reduced at Z‐discs in muscles from homozygous W2711X filamin‐C and heterozygous and homozygous R349P desmin knock‐in mice (Figure 2C–E″).

### 3.2 A Novel Approach to Quantify the Number and Size of Sarcomeric Lesions in Cross‐Sections of Skeletal Muscle Tissue

**FIGURE 3:**
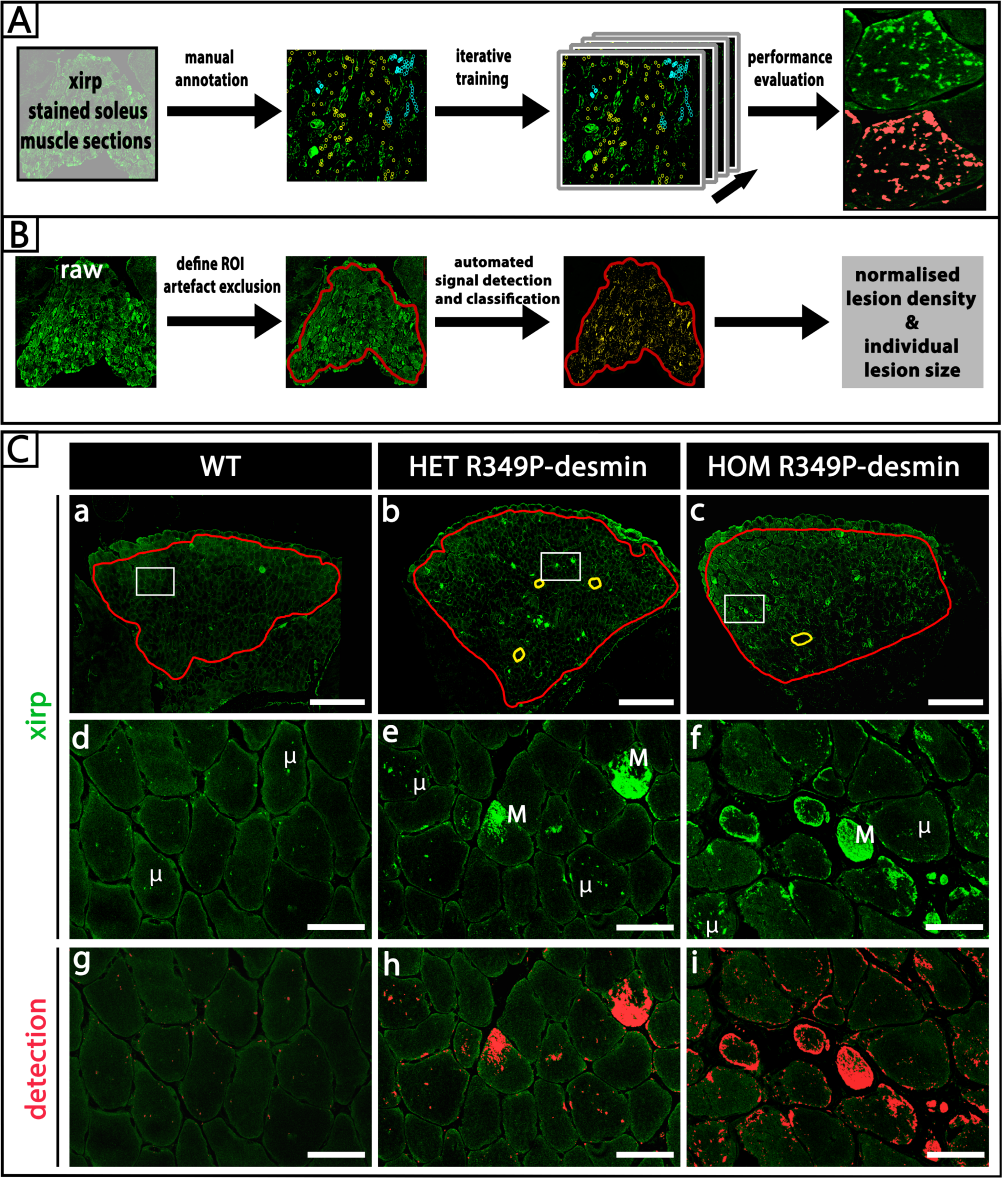
Software‐based, semi‐automatic detection of xirp 1/2‐positive myofibrillar lesions. (A) Scheme of the training process of a pixel classification algorithm using the open‐source software QuPath [29]. For this purpose, xirp 1/2 (xirp)‐positive sarcomeric lesions were manually annotated in immunofluorescence images derived from transverse sections of murine soleus muscle. Subsequently, annotated images were used for iterative training, and lesion recognition performance of the software was independently controlled by two expert investigators of the study. (B) Established and standardised analysis workflow for immunofluorescence staining of soleus muscle sections, image acquisition, the QuPath‐based manual annotation of regions of interest (ROI) and automatic identification and quantitation of sarcomeric lesions. (C) Representative images of lesions in xirp 1/2 (xirp) immunofluorescence stained cryosections (a–c, in green) and ROI annotations (red lines) for automated analysis in whole soleus muscle sections derived from wild‐type controls (left column) and heterozygous (middle column) and homozygous (right column) R349P desminopathy mice. Small areas with artefacts (small yellow circles in b and c) were manually annotated for their exclusion from analysis. White rectangles in (a–c) correspond to the magnified regions shown in (d–f). μ, multiple microlesions, M, macrolesions exceeding areas of 10 μm^2^. Detection, overlay of the xirp 1/2 (xirp) immunostain in green with the software‐identified sarcomeric lesions in red (g–i).

Based on the ability to detect sarcomeric lesions by xirp 1/2 immunofluorescence in cryosections, we next stained transverse muscle sections to systematically assess and quantify xirp 1/2‐positive sarcomeric lesions in cross‐sections of soleus muscle from R349P desmin knock‐in mice (Figure 3A,B). Here, xirp 1/2‐positive sarcomeric lesions appeared as small to larger sarcoplasmic spots and areas of fluorescence signal enrichment predominantly in muscles from heterozygous and homozygous R349P desmin knock‐in and to a lower extent also in muscles from wild‐type mice (Figure 3C,a–f). In the heterozygous and homozygous mice, soleus muscle fibres displayed a high degree of variability both in number and size of xirp 1/2‐positive lesions (Figure 3C,e,f); however, nearly all muscle fibres from homozygous soleus muscle contained xirp 1/2 signal enrichments (Figure 3C,f).

To facilitate a standardised quantitation of the number and size of xirp 1/2‐positive sarcomeric lesions in skeletal muscle tissue, we employed the open‐source digital pathology application QuPath ( [29], https://qupath.github.io), as described in detail in Section 2. Xirp 1/2‐positive sarcomeric lesions were manually annotated in multiple immunofluorescence images, and these processed images were used for iterative training of a QuPath binary pixel classification algorithm to distinguish between lesion and nonlesion areas (Figure 3A). The algorithm with the highest sensitivity in conjunction with the lowest rate of false‐positive detections was selected for final sarcomeric lesion quantification (Figure 3B). Thus, the final workflow for xirp 1/2 immunofluorescence‐based detection of sarcomeric lesions included staining of muscle sections, confocal image acquisition, QuPath‐based manual annotation of the soleus muscle as a ROI, manual exclusion of artefact‐containing regions (if any) and the automatic identification and quantitation of sarcomeric lesions (Figure 3C).

**FIGURE 4:**
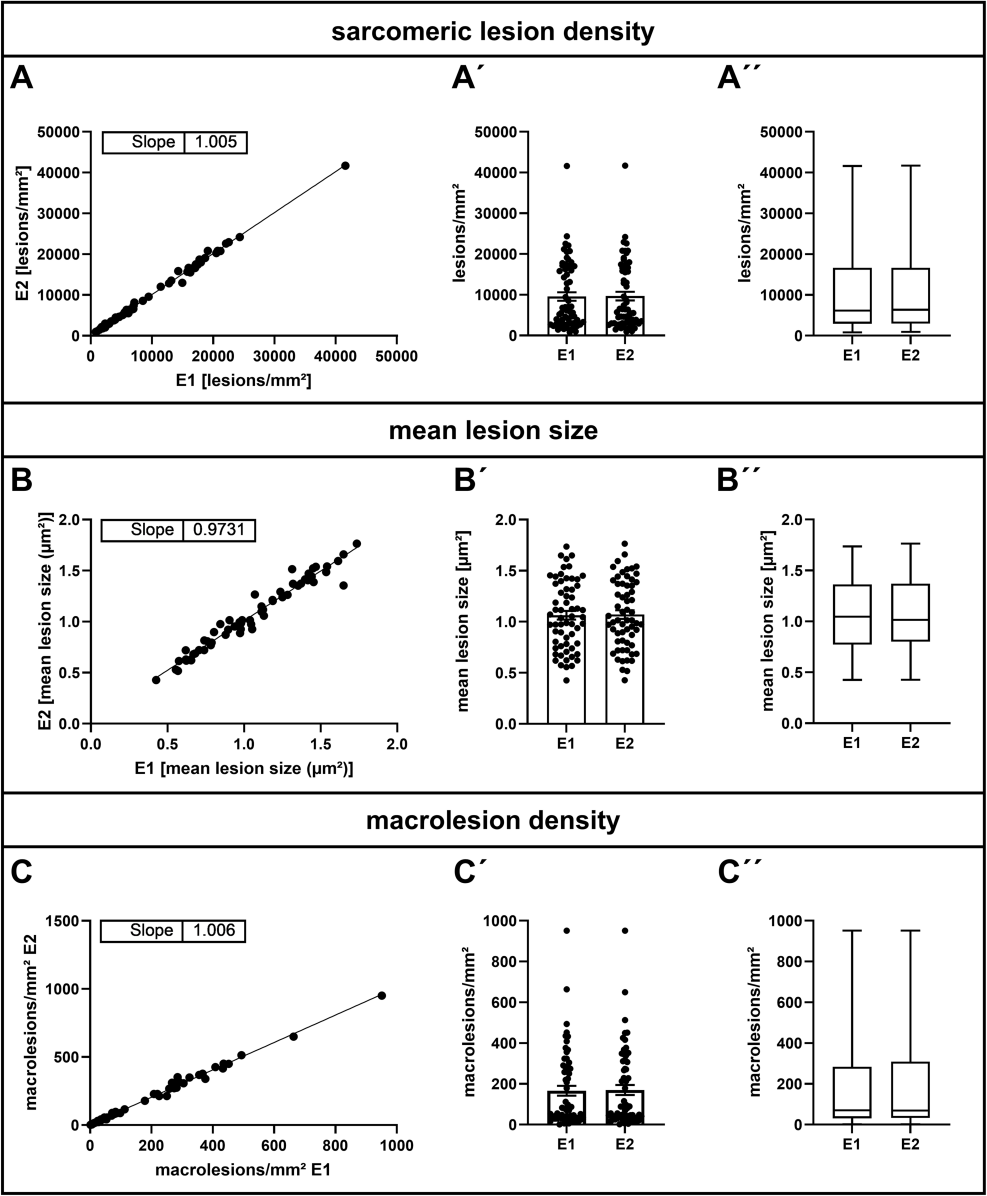
Exclusion of interobserver variability on the results of the software‐based sarcomeric lesion detection. Two independent investigators, E1 and E2, performed the software‐based analysis of sarcomeric lesions in a total of 63 stained samples (this work and other projects) from R349P desmin knock‐in mice and wild‐type littermates. (A–C) Correlation plots indicate no interobserver variability in the quantitation of lesion density (A), mean lesion size (B) and macrolesion density (C). (A´–C´) Scatter bar plots of lesion density (A´), mean lesion size (B´) and macrolesion density (C´) demonstrate negligible differences in mean values generated by E1 and E2. (A´´–C´´) Box plots demonstrating that not only the average values, but also the distribution of values within each dataset shows only minimal variance between the results of the two investigators.

To exclude potential interobserver variability, two independent investigators performed a blinded quantitative sarcomeric lesion analysis in an identical series of xirp 1/2 immunostained soleus sections derived from R349P desmin knock‐in mice. Detailed comparison of the data obtained allowed the exclusion of any statistically relevant interobserver variability of this software‐based sarcomeric lesion detection (Figure 4). Thus, this novel approach enables a highly reproducible sarcomeric lesion quantitation in large image data sets.

### 3.3 The Effect of Acute Strenuous Treadmill Exercise on the Sarcomeric Lesion Pathology in R349P Desmin Knock‐In Mice

**FIGURE 5:**
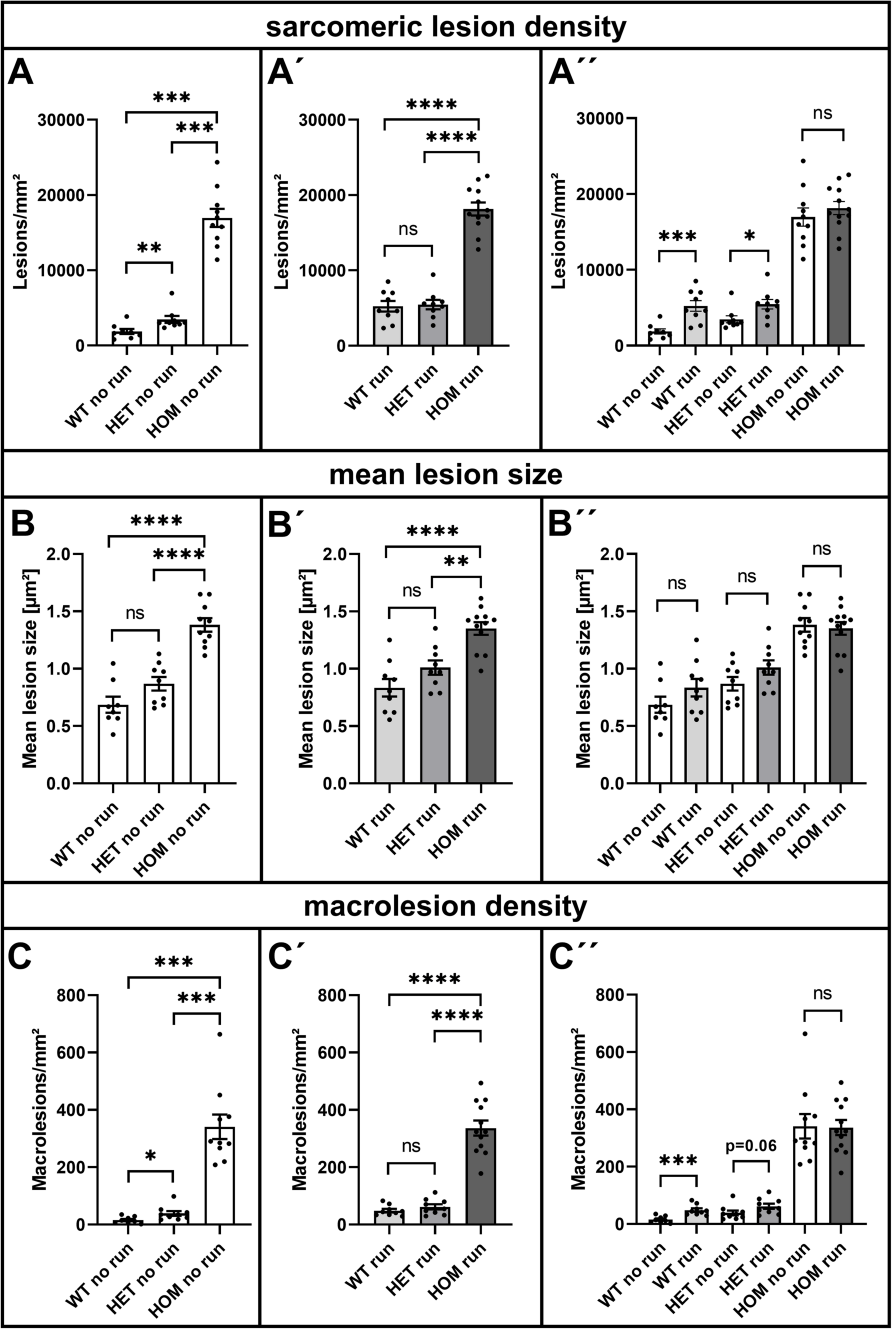
Quantitative analysis of xirp 1/2‐positive myofibrillar lesions in transverse sections of soleus muscle from sedentary and exercised R349P desminopathy mice. (A, A´, A´´) Sarcomeric lesion density in lesions per mm^2^ in soleus muscle from nonexercised (no run, A, A″) and strenuously exercised (run, A′, A″) heterozygous and homozygous R349P desmin knock‐in mice and wild‐type littermates. (A) Nonexercised heterozygous and homozygous animals showed statistically significantly more lesions per mm^2^ than the corresponding wild‐type. (A´) In exercised animals, no significant difference in lesion density was detected between wild‐type and heterozygous animals. (A´´) Compilation of lesion densities in sedentary and exercised animals. Note that exercise led to a statistically significant increase in lesion density in exercised wild‐type (*p* < 0.001) and heterozygous (*p* < 0.05) animals, whereas there was no significant increase in homozygous animals, which already had a high level of lesion density in the nonexercised state. (B, B´, B´´) Mean sarcomeric lesion size in μm^2^ in soleus muscle from nonexercised (no run, B, B″) and strenuously exercised (run, B′, B″) heterozygous and homozygous R349P desmin knock‐in mice and wild‐type littermates. (B, B′) In soleus muscle of nonexercised and exercised homozygous animals the mean lesion size was statistically significantly increased when compared to the corresponding heterozygous and wild‐type conditions. (B″) In none of the three genotypes did exercise lead to a significant increase in lesion size. (C, C´, C´´) Mean macrolesion density in lesions per mm^2^ in soleus muscle from nonexercised (no run, C, C″) and strenuously exercised (run, C′, C″) heterozygous and homozygous R349P desmin knock‐in mice and wild‐type littermates. These graphs show the proportion of lesions quantified in A‐A" that had a lesion area > 10 μm^2^. (C) Nonexercised heterozygous, and much more pronounced homozygous animals, showed statistically significantly more macrolesions per mm^2^ than the corresponding wild‐type. (C´) In exercised animals, no significant difference in macrolesion density was detected between wild‐type and heterozygous animals. (C´´) Compilation of macrolesion densities in sedentary and exercised animals in analogy to the graph in A″. Note that exercise led to significant increases in macrolesion density in exercised wild‐type (*p* < 0.001) and heterozygous (however, *p* = 0.06) animals, whereas there was no significant increase in homozygous animals, which already had a high level of macrolesion density in the nonexercised state. Sample size for each subgroup was: WT no run *n* = 8, WT run *n* = 9, Het no run *n* = 9, Het run *n* = 9, Hom no run *n* = 10, Hom run *n* = 12. For details of statistical analyses, please refer to the respective paragraph in the Materials section. All data are presented as mean ± SEM; *p < 0.05, **p < 0.01, ***p < 0.001, ****p < 0.0001.

To exploit the newly established workflow for the quantitation of sarcomeric lesions, we next studied the effect of acute strenuous treadmill exercise in heterozygous and homozygous R349P desmin knock‐in mice and wild‐type littermates. For this purpose, we analysed soleus muscles derived from the following six groups of mice: (i) nonexercised wild‐type (WT no run), *n* = 8; (ii) nonexercised heterozygous (HET no run), *n* = 9; (iii) nonexercised homozygous (HOM no run), *n* = 10; (iv) exercised wild‐type (WT run), *n* = 9; (v) exercised heterozygous (HET run), *n* = 9; and (vi) exercised homozygous (HOM run), *n* = 12. From each mouse, a representative xirp 1/2‐immunostained section containing the whole of the soleus muscle (see Section 2) was subjected to sarcomeric lesion analysis, which addressed the mean lesion density (lesions/mm^2^), the mean size of the lesions (mean lesion size in μm^2^) and the mean density of the subset of large sarcomeric lesions (macrolesions/mm^2^; > 10 μm^2^; spanning across multiple sarcomeres and adjacent myofibrils) (Figure 5). Soleus muscle sections from nonexercised wild‐type mice had a mean lesion density of 1874 lesions per mm^2^, whereas the samples from nonexercised heterozygous and homozygous R349P desmin knock‐in mice contained significantly higher numbers of 3473 and 16,924 lesions per mm^2^, respectively (Figure 5A). Acute treadmill exercise led to a significant increase in both wild‐type (5232 lesions per mm^2^) and heterozygous (5468 lesions per mm^2^) genotypes, with no significant difference between the two groups (Figure 5A′, A″). The already high lesion density in the nonexercised homozygous mice did not significantly increase following exercise (18,149 lesions per mm^2^) (Figure 5A″). The mean lesion size in the soleus muscle sections from nonexercised wild‐type, heterozygous and homozygous mice was approximately 0.7, 0.9, and 1.4 μm^2^, respectively. Here, only the homozygous tissue differed with statistical significance from the wild‐type and heterozygous genotypes (Figure 5B). Treadmill exercise did not significantly affect the mean lesion size (wild‐type, 0.8 μm^2^; heterozygous, 1.0 μm^2^; and essentially unchanged homozygous, 1.4 μm^2^) (Figure 5B′, B″). Macrolesions as a fraction of the overall lesions were rarely found in soleus muscle from nonexercised wild‐type mice (16 macrolesions per mm^2^). Heterozygous mice showed a significantly higher macrolesion density (39 macrolesions per mm^2^), while soleus muscle from nonexercised homozygous animals exhibited the highest value (341 macrolesions per mm^2^) (Figure 5C). Exercise again led to a significant increase in both wild‐type (49 macrolesions per mm^2^) and heterozygous (62 macrolesions per mm^2^, *p*‐value however 0.06) mice, with no significant difference between the two groups, while the macrolesion density in the exercised homozygous genotype remained essentially unchanged (337 macrolesions per mm^2^) (Figure 5C′, C″).

## 4 Discussion

### 4.1 Xin Actin‐Binding Repeat‐Containing Proteins and Filamin‐C Are Markers for Myofibrillar Damage in Skeletal Muscle Tissue

Extending our previous work on W2711X filamin‐C knock‐in mice [16, 17], we here demonstrate the presence of xin actin‐binding repeat‐containing proteins 1 and 2 (xirp 1 and xirp 2) and filamin‐C‐positive sarcomeric lesions in longitudinal sections of soleus muscles derived from mouse models for human desminopathies, comprising heterozygous and homozygous R349P [24] and R405W [25, 26] desmin knock‐in mice as well as desmin knock‐out mice [27, 28]. These sarcomeric lesions, which were also present in nonexercised and to a greater extent in exercised wild‐type siblings, are likely the equivalent of areas with Z‐disc streaming at the ultrastructural level in response to physical exercise and eccentric contractions in human skeletal muscle [35]. Our findings show that immunolocalisation of xirp 1/2 and filamin‐C can very reliably visualise myofibrillar damage at the light microscope level in skeletal muscle tissue derived from nonexercised and exercised wild‐type, desminopathy and filaminopathy mice.

### 4.2 A Novel Approach to Quantify Sarcomeric Lesions at the Light Microscope Level

Since a reliable ultrastructural quantification of myofibrillar damage is currently almost impossible in larger sections of skeletal muscle tissue, we set out to develop a routine tool to quantify sarcomeric lesions in cross‐sections of skeletal muscle, which are used in routine myopathological diagnostics. For this purpose, we established a digital image analysis protocol enabling the detection and measurement of lesions in cross‐sections of normal and diseased skeletal muscle tissue specimens stained with an antibody directed against xirp 1/2. The outputs were quantified values for ‘sarcomeric lesion density’ in number of lesions per mm^2^, ‘mean lesion size’ in μm^2^ and ‘macrolesion density’ in macrolesions per mm^2^. The established semi‐automatic workflow kept the necessary training effort and computing power to a minimum, while achieving satisfactory detection performance with robust interrater reliability. To obtain reliable data sets from analysing a series of muscle sections based on the same trained pixel classifier, all the parameters of the immunofluorescence staining procedure, microscopy settings and image acquisition mode have to be kept constant.

### 4.3 Sarcomeric Lesion Formation in R349P Desmin Knock‐In Mice in Response to Mechanical Strain

Using our newly established workflow for quantifying xirp 1/2‐positive sarcomeric lesions, we investigated the effect of acute mechanical strain on the myofibrillar apparatus in R349P desmin knock‐in mice. These mice are a well‐established disease model for human R350P‐related desminopathy [24, 36] and exhibit structural and functional alterations of the extrasarcomeric cytoskeleton [37, 38]. Our quantitative analysis revealed significant differences between nonexercised mice across the three genotypes, with a moderately higher mean lesion density in heterozygous and a more than threefold higher density in homozygous R349P desmin knock‐in mice. After a single bout of strenuous treadmilling, both wild‐type and heterozygous R349P desmin knock‐in mice showed a significant increase in the density of sarcomeric lesions, whereas in nonexercised homozygous mice, the lesion density was already high and did not further increase upon exercise. Given the already very high mean lesion density in homozygous mice even under sedentary conditions, we concluded that the observed effect of strenuous exercise had reached the top end of the flagpole of maximum lesion formation in this genotype already without additional mechanical stress.

Differences between the three genotypes in nonexercised and exercised mice were also observed for mean lesion size, which was consistently the lowest in wild‐type and highest in homozygous mice but showed no effect of treadmilling. Likewise, the extent of macrolesions differed in a genotype‐specific manner under basal conditions, with the lowest values in wild‐type animals and highest values in homozygous mice. In this case, treadmilling also led to an increase in macrolesion density in wild‐type and heterozygous mice, but not in homozygous mice, again implying that lesion formation is at its maximal level even in the absence of elevated mechanical stress. Previous work, in which xirp 1/2‐positive sarcomeric lesions were quantified in heterozygous W2711X filamin‐C knock‐in mice after a single bout of strenuous treadmilling, showed increased numbers of macrolesion‐containing muscle fibres but no increase in the number of fibres with microlesions. In these mice, however, there was a broadening of the range to a markedly higher number of fibres with microlesions (Figure 6B in [16]). Although the results between the W2711X filamin‐C knock‐in and the R349P desmin knock‐in mice are not directly comparable due to methodological differences, our findings on myofibrillar lesion formation suggest that both R349P mutant desmin and W2711X mutant filamin‐C exert deleterious effects on the myofibrillar apparatus as a key pathogenic step in these two forms of myofibrillar myopathies.

### 4.4 Pathogenetic Significance of Sarcomeric Lesions in Desminopathies

Mechanical strain‐induced sarcomeric lesions are focal Z‐disc disruptions that occur in both human and murine skeletal muscle [20, 35]. They constitute a physiological phenomenon that makes muscle fibres less vulnerable to subsequent strenuous exercise [35]. It is thought that this adaptation reduces the number of stress‐sensitive fibres, thereby limiting the severity of myofibrillar damage that could eventually lead to prolonged or even permanent muscle weakness [20, 35]. The elevated number of xirp 1/2‐positive lesions in R349P desmin knock‐in mice suggests that the increased occurrence of this physiological adaptation process reflects an underlying intrinsic problem of mechanical stability, stress resistance or maintenance of myofibrillar Z‐discs. This problem is associated with a structurally and functionally compromised extrasarcomeric desmin cytoskeleton in the desminopathy animals. The drastically higher lesion burden in the homozygous R349P desmin mice also highlights that the total disruption of the desmin cytoskeleton in these animals [24, 37] impairs myofibrillar stability more severely than the focal derangements induced by the mixed expression of wild‐type and mutant desmin in heterozygous animals [24]. This also mirrors clinical findings in patients with autosomal‐dominant versus autosomal recessive desminopathy, where the latter have an earlier disease onset in conjunction with a more severe and rapidly progressive myopathy and cardiomyopathy [39]. The results of our treadmill experiments in R349P desmin knock‐in mice indicate that even single bouts of high‐intensity physical exercise may aggravate a preexisting myofibrillar pathology, creating an even greater demand for myofibrillar repair processes. Additional studies are needed to address the repair of the exercise‐induced sarcomeric lesions over time, as well as the myofibrillar integrity and force development in the context of desminopathies and other myofibrillar myopathies.

## Author Contributions

R.S. and C.S.C. conceived the study, designed experiments and reviewed all data. C.H. and D.S. carried out experiments, developed protocols, analysed data, designed figures and drafted the manuscript. C.B., J.S. and C.S.C. carried out experiments, developed protocols and analysed data. U.S.S. and M.R.N. carried out experiments and analysed data. S.B.P., A.L., E.M., P.F.M.v.d.V. and D.O.F. provided materials, developed protocols and analysed data. C.H., D.S., R.S. and C.S.C. performed statistical evaluations. C.H., D.S., D.O.F., R.S. and C.S.C. jointly finalised the manuscript text and figures. All authors have read and agreed to the final version of the manuscript.

## Ethics Statement

All procedures involving mice as described in Section 2 were approved by the responsible authorities, the governmental office for animal care (Landesamt für Natur, Umwelt und Verbraucherschutz North Rhine‐Westphalia (LANUV NRW), Recklinghausen, Germany (reference number 84‐02.04.2016.A421) and the University Paris Diderot local committee (authorisation number CEB‐16‐2016/2016041216476300).

## Conflicts of Interest

The authors declare no conflicts of interest.

## Supporting information


**Table S1:** List of all mice and data obtained by sarcomeric damage quantification. Excel file listing the data for the individual mice in the six groups of animals (wild‐type, heterozygous, homozygous; no run control, run). Columns B–E, mouse ID, genotype, sex, age. Column F, no run control or run condition. Columns G–M, values obtained by image analysis and sarcomeric damage quantification (region of interest, number of detections, lesions per mm^2^, mean lesion size, number of macrolesions, macrolesions per total lesions, macrolesions per mm^2^).

**Figure S1:** **Confirmation of xirp immunodetection**. Western blot of protein extracts from differentiated C2 mouse myoblasts incubated with the rabbit polyclonal antiserum against xin actin‐binding repeat‐containing proteins 1 and 2 (xirp 1, xirp 2) (antiserum #7700; see Materials and Methods section and [19]). In control cell extracts, the antiserum recognised three bands representing xirp1a and xirp1b (calculated molecular mass 196.7 and 123.4 kDa, respectively) and xirp2 (428.3 kDa). Transfection of these cells with an siRNA against xirp1a and xirp1b specifically reduced the expression of both xirp1 isoforms but not of xirp2. Note that all xirps run slower than their predicted molecular mass.

**Data S1:** Supporting Information.

## Data Availability

The data that support the findings of this study are available within the article.
