# Supplementary figures and images for: Quantification of Exercise‐Induced Sarcomeric Damage in R349P Desmin Knock‐In Mice: A New Approach in Myofibrillar Myopathy Research
Source: Neuropathol Appl Neurobiol. 2025 Sep 14;51(5):e70038. doi: 10.1111/nan.70038 (PMC12433831; doi:10.1111/nan.70038)

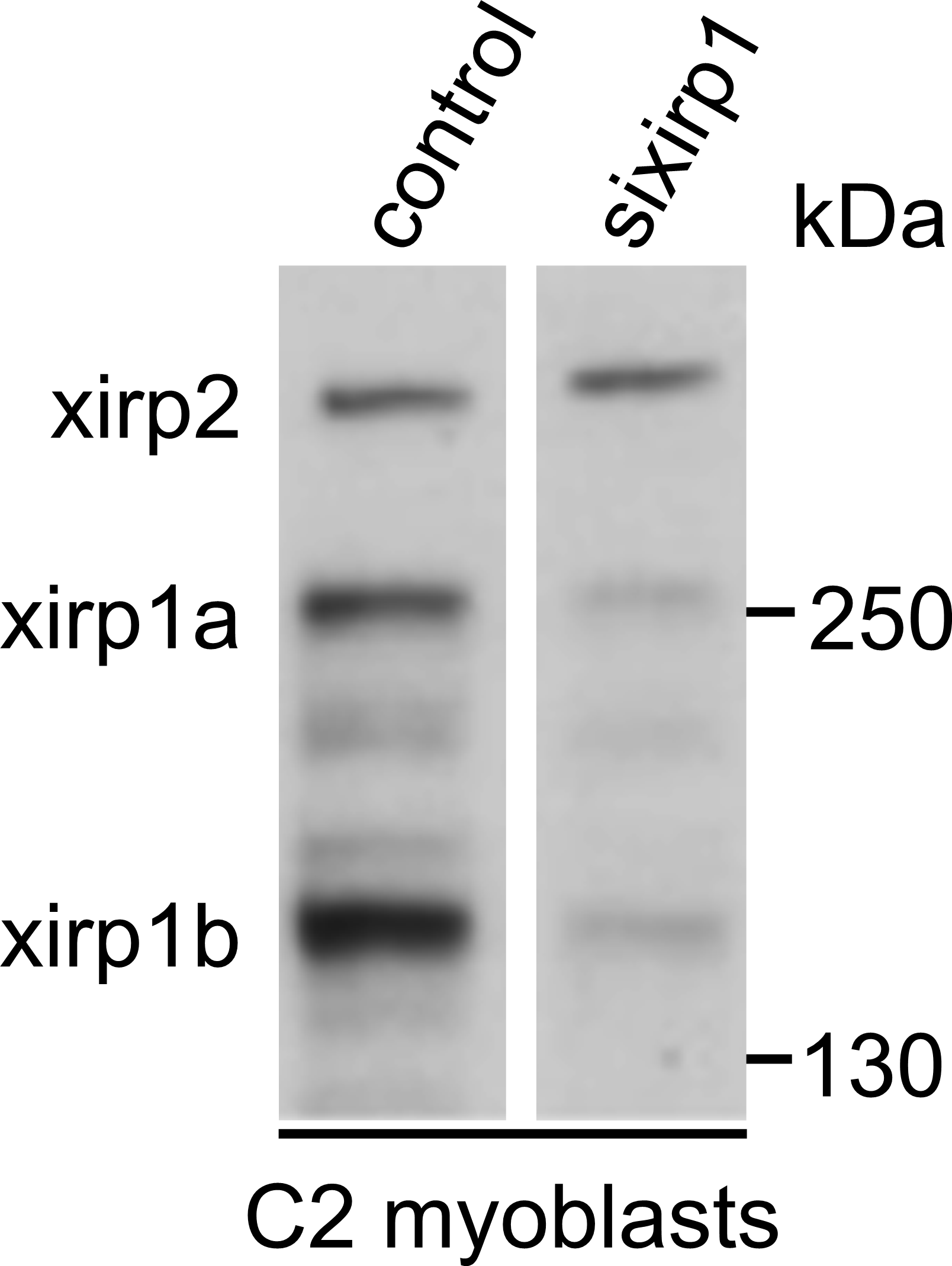

Supplement: Supplementary file 2 — Figure S1: Confirmation of xirp immunodetection. Western blot of protein extracts from differentiated C2 mouse myoblasts incubated with the rabbit polyclonal antiserum against xin actin‐binding repeat‐containing proteins 1 and 2 (xirp 1, xirp 2) (antiserum #7700; see Materials and Methods section and [19]). In control cell extracts, the antiserum recognised three bands representing xirp1a and xirp1b (calculated molecular mass 196.7 and 123.4 kDa, respectively) and xirp2 (428.3 kDa). Transfection of these cells with an siRNA against xirp1a and xirp1b specifically reduced the expression of both xirp1 isoforms but not of xirp2. Note that all xirps run slower than their predicted molecular mass. [file NAN-51-e70038-s001.tif]
