# Supplementary material for: Quantification of Exercise‐Induced Sarcomeric Damage in R349P Desmin Knock‐In Mice: A New Approach in Myofibrillar Myopathy Research
Source: Neuropathol Appl Neurobiol. 2025 Sep 14;51(5):e70038. doi: 10.1111/nan.70038 (PMC12433831; doi:10.1111/nan.70038)
Supplement: Supplementary file 3 — Data S1: Supporting Information. [file NAN-51-e70038-s003.docx]

**Quantification of exercise-induced sarcomeric damage in R349P desmin knock-in mice:
a new approach in myofibrillar myopathy research**

Christian Holtzhausen^1^, Dorothea Schultheis^1^, Carolin Berwanger^2,3^, Julia Schuld^4^, Ursula Schlötzer-Schrehardt^5^, Marion Riehl-Nestler^6^, Sabrina Batonnet-Pichon^7,8^, Alain Lilienbaum^7^, Esther Mahabir^6^, Peter F. M. van der Ven^4^, Dieter O. Fürst^4^, Rolf Schröder^1,*,#^, Christoph S. Clemen^2,3,*,#^

^1^ Institute of Neuropathology, Universitätsklinikum Erlangen, Friedrich-Alexander-Universität Erlangen-Nürnberg (FAU), 91054 Erlangen, Germany

^2^ Institute of Aerospace Medicine, German Aerospace Center, 51147 Cologne, Germany

^3^ Institute of Vegetative Physiology, Medical Faculty, University of Cologne, 50931 Cologne, Germany

^4^ Institute for Cell Biology, University of Bonn, 53121 Bonn, Germany

^5^ Department of Ophthalmology, Universitätsklinikum Erlangen, Friedrich-Alexander-Universität Erlangen-Nürnberg (FAU), 91054 Erlangen, Germany

^6^ Comparative Medicine, Center for Molecular Medicine Cologne, Faculty of Medicine and University Hospital Cologne, 50931 Cologne, Germany

^7^ Basic and Translational Myology, Unit of Functional and Adaptive Biology, Université Paris Cité / CNRS UMR 8251, Paris, France

^8^ Institut Cochin, Université Paris Cité, INSERM U1016, CNRS, Paris, France

^*^These two senior authors contributed equally to this work.

^#^Authors for correspondence:

Prof. Dr med. Rolf Schröder, Institute of Neuropathology, Universitätsklinikum Erlangen, Schwabachanlage 6, 91054 Erlangen, Germany;

Phone: +49 9131 85 34782; Email: rolf.schroeder@uk-erlangen.de

Prof. Dr med. Christoph S. Clemen, Institute of Aerospace Medicine, German Aerospace Center, Linder Höhe, 51147 Cologne, Germany;

Phone: +49 2203 601 3468; Email: christoph.clemen@uni-koeln.de

**Supplementary Legends**

**Supplementary Table 1.** **List of all mice and data obtained by sarcomeric damage quantification.** Excel file listing the data for the individual mice in the six groups of animals (wild-type, heterozygous, homozygous; no run control, run). Columns B to E, mouse ID, genotype, sex, age. Column F, no run control or run condition. Columns G to M, values obtained by image analysis and sarcomeric damage quantification (region of interest, number of detections, lesions per mm², mean lesion size, number of macrolesions, macrolesions per total lesions, macrolesions per mm²).

**Supplementary Figure 1.** **Confirmation of xirp immunodetection.** Western blot of protein extracts from differentiated C2 mouse myoblasts incubated with the rabbit polyclonal antiserum against xin actin-binding repeat-containing proteins 1 and 2 (xirp 1, xirp 2) (antiserum #7700, see Materials and Methods section and [19]). In control cell extracts, the antiserum recognised three bands representing xirp1a and xirp1b (calculated molecular mass 196.7 and 123.4 kDa, respectively) and xirp2 (428.3 kDa). Transfection of these cells with an siRNA against xirp1a and xirp1b specifically reduced the expression of both xirp1 isoforms, but not of xirp2. Note that all xirps run slower than their predicted molecular mass.

[19] Kebir S, Orfanos Z, Schuld J, Linhart M, Lamberz C, van der Ven PFM, et al. Sarcomeric lesions and remodeling proximal to intercalated disks in overload-induced cardiac hypertrophy. Exp Cell Res 2016; 348: 95-105
